# Does perceived ostracism contribute to mental health concerns among veterans who have been deployed?

**DOI:** 10.1371/journal.pone.0208438

**Published:** 2018-12-06

**Authors:** Eric D. Wesselmann, Dan Ispas, Mark D. Olson, Mark E. Swerdlik, Natasha M. Caudle

**Affiliations:** 1 Department of Psychology, Illinois State University, Normal, Illinois, United States of America; 2 School of Social Work, Illinois State University, Normal, Illinois, United States of America; Stellenbosch University, SOUTH AFRICA

## Abstract

Posttraumatic stress–negative psychological experiences as a result of traumatic stressors–can hinder military veterans’ reintegration into society and cause various mental health problems. Veterans need quality social relationships to facilitate reintegration and to cope with posttraumatic stress and related mental health problems; discrimination or other forms of interpersonal rejection can exacerbate these veterans’ problems. Ostracism (i.e., being ignored and excluded) is a painful and psychologically distressing experience that may be one factor that contributes to the problems of veterans who are dealing with posttraumatic stress. To our knowledge, this connection has yet to be tested empirically. Thus, we investigated the correlation between posttraumatic stress, perceived ostracism, and other theoretically relevant variables (i.e., mental health problems, perceived social support, psychological need satisfaction) in a sample of veterans who have had at least one deployment. Our results provide preliminary empirical evidence suggesting that perceived ostracism may contribute to veteran’ deployment-related psychological problems. Veterans’ perceived ostracism correlated with psychological problems (i.e., posttraumatic stress symptoms, anxiety and psychological distress), and it explained additional variance in posttraumatic stress symptoms above and beyond common predictors of these symptoms (i.e., deployment stress, perceived military and civilian-based social support). Finally, perceived ostracism emerged as the most important predictor of posttraumatic stress symptoms in a relative weights analysis.

## Introduction

Military veterans often face problems reintegrating into civilian life after being deployed, including posttraumatic stress–negative psychological experiences resulting from traumatic stress [1]. Individuals suffering from posttraumatic stress typically exhibit symptoms involving cognitively re-experiencing the stressful event, emotional numbness or dysphoria, feelings of hyperarousal, and effortful avoidance of reminders of the stressful event [2–3]. In general, the more combat-related stressors veterans experienced during deployment, the more likely they are to experience posttraumatic stress symptoms post-deployment [1, 4].

Posttraumatic stress often co-occurs with other mental health problems, such as depression [5–7], anxiety [6, 8], substance use [5,7], aggressive behavior [9–10], and suicide [11–12]. The availability and quality of social support (from fellow soldiers, civilian contacts, family members, and even strangers) can influence veterans’ recovery from posttraumatic stress and related mental health problems [11, 13–15]. Social support, broadly defined as behaviors (whether formal or informal) focused on helping someone who perceived to be in need of help [16–17] is an important part of one’s overall feelings of belonging in their social relationships [18].

However, veterans often experience stigmatization and interpersonal rejection; these threats to their relationships can prolong distress and discourage them from seeking help [7, 19–21]. McGraw [22] recently suggested that a particular threat to social relationships–*ostracism* (i.e., being ignored and excluded [23])–may contribute to veterans’ poor mental health outcomes post-deployment and encouraged future empirical research to consider this possibility. Considerable research exists on ostracism in civilian samples; these events are distressing, even when they are innocuous and brief [23–24]. Research suggests ostracism may be a unique social threat in that it is characterized by feeling *ignored* or *invisible* in one’s relationships [23]. There are various ways that people can experience these feelings in their relationships, many of which are subtle or ambiguous. For example, individuals can feel ostracized when they do not receive text messages [25], social media feedback [26], and even eye contact [27–28]. Further, ostracism typically hurts regardless of whether it is perpetrated by close friends/relatives or by strangers [29].

Both correlational and experimental research suggest ostracism can cause various physical and psychological problems, such as increased cardiovascular problems and heightened cortisol levels [30–31], and various negative emotions (e.g., anger, sadness, shame; [23, 32–33]). Further, ostracism can increase individuals’ tendencies to ruminate on negative events [34] and behave aggressively towards others [35]. Ostracism can even make individuals feel dehumanized [36] and contribute to a sense that life is generally meaningless [37].

Ostracism threatens core psychological needs that are often satisfied by having stable and supportive interpersonal relationships: the need for *belonging*, *self-esteem*, *meaningful existence*, and *control* [38]. Generally, individuals should have these needs satisfied to maintain a sense of overall personal security; thus instances of ostracism represent a serious threat to one’s overall well-being [39]. Further, individuals who experience chronic ostracism or otherwise feel lonely can experience extreme outcomes, such as feelings of alienation, unworthiness, depression, helplessness, and increased mortality rates [40–42].

### Current research

To our knowledge, no previous study has examined McGraw’s [22] suggestion that ostracism may contribute to the negative psychological effects deployment-related stress can have on veterans. However, given the various negative psychological and physical outcomes for ostracism in civilian samples, it is a reasonable hypothesis. Before testing this hypothesis, we first wanted to replicate basic findings from previous literature on deployment stress, posttraumatic stress symptoms, and related mental health outcomes. In general, the more deployment-related stress veterans experienced, the more likely they are to experience posttraumatic stress symptoms post-deployment [1, 4]. Additionally, posttraumatic stress is comorbid with other types of psychological distress experienced post-deployment (e.g., anxiety and depression; [6, 8]). Finally, research demonstrates that veterans who perceive having positive social support (from military or civilian sources) have more positive psychological outcomes than veterans who do not [14–15]. Thus, we hypothesize the following:

*Hypothesis 1a* –Veterans’ recalled deployment stress will correlate positively with posttraumatic stress symptoms.
*Hypothesis 1b* –Veterans’ posttraumatic stress symptoms will correlate positively with other negative psychological outcomes.
*Hypothesis 1c* –Veterans’ perceived social support will correlate negatively with posttraumatic stress symptoms and other negative psychological outcomes.

Next, we wanted to test the theorized links between deployment stress, mental health, and perceived ostracism [22]. Given that ostracism involves feeling both ignored and excluded from social interactions [23], veterans who perceive a lack of social support likely feel ostracized in their interpersonal relationships and by their larger organizational affiliations (such as their unit or the military broadly [22]).

*Hypothesis 2a* –Veterans’ perceived ostracism will correlate positively with posttraumatic stress symptoms and other negative psychological outcomes (i.e., anxiety and psychological distress).
*Hypothesis 2b* –Veterans’ perceived ostracism will correlate negatively with perceived social support (both from civilian and military sources).

When examining a new predictor, it is common to examine the incremental variance it explains over other, well-established predictors. This is usually accomplished using hierarchical multiple regression. Tonidandel and LeBreton [43] also recommend examining the relative importance of the predictors, which allows for examining the impact of a predictor relative to others in the model. Therefore, we will also examine, in an exploratory fashion, the relative importance and incremental variance explained by perceived ostracism in predicting posttraumatic stress symptoms while taking into account the two types of perceived support (civilian and military) and deployment stress.

*Research question 1a* –Does perceived ostracism explain incremental variance in posttraumatic stress symptoms over deployment stress and perceived support?
*Research question 1b* –What is the relative importance of perceived ostracism, deployment stress, perceived military support and civilian support for posttraumatic stress symptoms?

## Materials and methods

### Participants

This study was approved by the IRB at Illinois State University. We collected a sample of veterans by sending recruitment emails (with an anonymous survey link) over listservs moderated by the Illinois Army National Guard. We offered participants a 20.00 USD digital gift card for Amazon.com as compensation. Interested participants (*N* = 129; 202 participants began the survey, but we only analyzed the data from participants who indicated having been previously deployed) completed the survey on their own time at the computer of their choice. Upon completing the survey, participants received debriefing information and links to online mental health resources; we also provided them with a separate survey link where they could supply their email to claim compensation. We stored the emails separate from the questionnaire responses to preserve anonymity.

Our sample included 103 men and 26 women (*M*_age_ = 36.66 years, *SD* = 8.41; 82.8% identified as White, 6.3% multi-racial, 3.1% Black/African American, 1.6% American Indian/Alaskan Native, 1.6% Asian, and 2.3% “other or unknown”; 5.5% also identified as Hispanic or Latino/a). In terms of education level, 66.4% of our sample had an Associate’s Degree or higher. In terms of relationship status, 70.9% of our sample were married or in legal partnerships. We did not assess the specific dates of deployment, but we did assess the number of deployments. Our sample reported an average of 1.75 (*SD* = .96) deployments. We also assessed participants’ current veteran status (see Table 1). One participant stopped after completing the demographics. Thus, we have not included this person’s information in any of the analyses.

**Table 1:** Participant demographic information.

Demographic Category	Response Option	*N*	Demographic Category	Response Option	*n*
Gender			Have Children?		
	Male	103		Yes	90
	Female	26		No	39
	Transgender	0	Number of Deployments		
	Prefer Not to Answer	0		1	65
Race				2	41
	American Indian or Alaskan Native	2		3	18
	Black or African-American	4		4	3
	White	107		5	0
	Native Hawaiian or other Pacific Islander	0		6 or More	2
	Asian	2	Highest Education Level		
	More Than One Race	8		No high school diploma	0
	Other or Unknown	3		High school diploma	5
	*Unanswered*	3		Some college	39
Hispanic or Latino(a)?				Associates Degree (AA)	30
	Yes	7		Bachelors Degree (BA or BS)	36
	No	100		Masters Degree	16
	*Unanswered*	22		Doctoral Degree	2
Marital Status				“Other” (i.e., post baccalaureate)	1
	Single	23	Veteran Status		
	Married/Legal Partnership	90		Active Duty	52
	Divorced	14		Reserve	57
	Separated	1		Retired Military	5
	“Other” (i.e., engaged)	1		No Longer in the Military but Not Formally Retired	15

Note: Total *N* = 129

### Procedure & measures

Participants first completed basic demographics (e.g., gender, race, age, deployment history). They then completed several measures in a standardized order (items within each measure were randomized). For all measures, we coded and averaged items together such that higher scores indicated higher endorsement of each construct.

#### Deployment stress

We used the Deployment Concerns subscale of the Deployment Risk and Resiliency Inventory-2 (DRRI-2 [44]) to assess participants’ experience with deployment stress. The Deployment Concerns subscale uses 12 items asking participants to indicate their agreement with various statements (e.g., “I thought I would never survive.”; 1-Strongly Disagree, 5-Strongly Agree; Cronbach’s α = .90). We asked participants to answer each question based upon their most recent deployment.

#### PTSD symptoms

We used the PTSD Checklist for DSM-5 (PCL-5 [45]) to assess participants’ self-reported posttraumatic stress symptoms. The PCL-5 uses 20 items based on the updated diagnostic criteria from the DSM-5 (e.g., “In the past month, how much were you bothered by repeated, disturbing dreams of the stressful experience?”; 1-Not At All, 5-Extremely; Cronbach’s α = .96). We used the standard instructions which do not anchor the items to a specific traumatic event, but rather asks participants to answer each item within the context of how they have felt within the past month.

#### Anxiety and psychological distress

We measured *anxiety* with the State-Trait Anxiety Inventory-Anxiety *trait* subscale; we selected the seven items specific to anxiety (rather than the items measuring comorbid depression symptoms) [46]. Participants indicated their general agreement with each item (e.g., “I worry too much over something that really doesn’t matter.”; 1-Not At All, 7-Very Much; Cronbach’s α = .94). We also used a measure of *state* anxiety (indicating their agreement based on how they felt at that moment) [47]; this measure was highly correlated with trait anxiety (*r* = .70, *p* < .001) and the pattern of results were largely the same. Thus we have omitted these results from the manuscript (available as supplementary material at https://osf.io/sb9z7/). We measured *psychological distress* using five items selected from the Mental Health Inventory [48]; these items have been used previously to assess psychological health in a military sample [49]. Participants indicated their agreement with each item based on how they typically felt within the past month (1-All of the time, 6-Never; Cronbach’s α = .92).

#### Perceived social support

We measured two aspects of perceived social support–one from civilian sources and one from military sources. For *civilian*-based perceived social support, we used the Post Deployment Social Support subscale of the Deployment Risk and Resiliency Inventory (DRRI [50]) to assess participants’ perceived social support from civilian sources. The Social Support subscale uses 15 items asking participants to indicate their agreement with various statements (e.g., “I am carefully listened to and understood by family members or friends.”; 1-Strongly Disagree, 5-Strongly Agree; Cronbach’s α = .90). We asked participants to answer each question based upon their most recent deployment.

We measured *military*-based perceived social support by having participants indicate their agreement with 14 items assessing how much support they perceived the military provided them and their families (e.g., “I feel encouraged by the military community to seek help for dealing with negative feelings (such as feeling “down”, anxious, or irritable).” We used the four items used in Smith et al. [15], and generated ten additional items to address sources of military-based support not covered in the original items (e.g., military chaplain, military-sponsored support groups). Some of these additional items assessed veterans’ perceptions of support available to their families, because family support resources can also influence veterans’ reintegration [51]. Participants answered all questions on a 5-point rating scale (1-Strongly Disagree, 5-Strongly Agree).

Because we created additional items beyond the previously established ones, we conducted an exploratory factor analysis on the military-based social support items using Principle Axis Factoring and Promax rotation with Kaiser Normalization. The analysis yielded two factors: Factor 1 had an eigenvalue of 7.65 and explained 54.6% of the variance, and Factor 2 had an eigenvalue of 1.69 and explained 12.0% of the variance. The pattern of items suggest that Factor 1 focused mostly on perceived accessibility and official encouragement for soldiers to use military-supported resources (all items we generated), and Factor 2 focused on perceived available social support from fellow veterans. However, we chose to combine these into one overall measure because both factors correlated at .65, Cronbach’s α = .93, and many of the items cross-loaded on each factor (see https://osf.io/sb9z7/ for full items and factor loadings).

#### Perceived ostracism and psychological need satisfaction

Participants indicated how much they perceived being ostracized in their daily lives; they indicated their agreement with ten items depending upon how often the events described happen to them generally [52–53]. They answered each item using a 7-point scale (1-Hardly Ever; 7-Almost Always; e.g., “In general, I feel ignored” and “In general, others treat me as if I’m in solitary confinement”; Cronbach’s α = .96).

The perceived ostracism measure is relatively new and has not been used with a military sample; as such we wanted to verify that individuals who reported experiencing more ostracism in their daily lives also reported lower basic psychological need satisfaction. We adapted the 20-item *psychological need satisfaction* measure typically used as a dependent variable in experimental research on ostracism [23]; we asked participants to indicate their agreement with each item based on how they typically feel on an average day, using a 5-point scale (1-Not at all; 5-Extremely). These items assess feelings of belonging, self-esteem, meaningful existence, and control; these needs can be examined separately or as one overall aggregate need satisfaction measure [23]. We had no hypotheses about each separate need so we coded and averaged all items together (Cronbach’s α = .96).

## Results and discussion

### Preliminary replications of past research

Before investigating our primary hypotheses and research questions, we conducted preliminary bivariate correlations to examine if the correlations among variables in our sample replicate correlations in previous literature on deployment stress, posttraumatic stress symptoms, and mental health outcomes (see Table 2 for all bivariate correlations). Replicating past research [1, 4], we found that veterans’ recalled deployment stress correlated positively with posttraumatic stress symptoms (*r* = .39, *p* < .001). Veterans’ posttraumatic stress symptoms also correlated positively with anxiety (*r* = .76, *p* < .001) and psychological distress (*r* = .73, *p* < .001). Finally, veterans’ perceived social support correlated negatively with posttraumatic stress symptoms and measures of anxiety and psychological distress. These patterns held for both civilian- (*ps* < .001) and military-based social support (*ps* < .01). Thus, our data supported Hypotheses 1a-c.

**Table 2:** Descriptive statistics, inter-correlation matrix, and scale reliabilities.

	Mean	SD	1	2	3	4	5	6	7	8
1. Psychological Distress	2.46	1.07	*(*.*92)*							
2. Perceived Ostracism	1.81	1.21	.63***	*(*.*96)*						
3. Basic Needs Satisfaction	3.80	.82	-.73***	-.78***	*(*.*96)*					
4. Trait Anxiety	2.45	1.47	.76***	.61***	-.73***	*(*.*94)*				
5. PTSD Symptoms	.90	.84	.73***	.65***	-.73***	.76***	*(*.*96)*			
6. Perceived Civilian-Based Social Support	3.86	.71	-.53***	-.55***	.59***	-.46***	-.51***	*(*.*90)*		
7. Deployment Stress	2.56	.92	.26**	.25**	-.33***	.40***	.39***	-.18*	*(*.*90)*	
8. Perceived Military-Based Social Support	3.85	.87	-.46**	-.54***	.64**	-.46***	-.56***	.60***	-.28**	*(*.*93)*

*p < .05.

**p < .01.

***p < .001.

*N*s = 125–128.

### Perceived ostracism and deployment-related measures

Hypothesis 2a focused on providing empirical support for a link between veterans’ perceived ostracism and deployment-related psychological problems. First, we found that perceived ostracism correlated positively with posttraumatic stress symptoms, anxiety, and psychological distress (*r*s ≥ .61, *ps* < .001). We also found that individuals who reported more perceived ostracism also reported less basic need satisfaction (*r* = -.78, *p* < .001), replicating typical effects found in laboratory manipulations of ostracism [23], and previous research using survey measures of ostracism in civilian samples [42]. Hypothesis 2b focused on the connection between perceived ostracism and various sources of perceived social support. As expected, veterans’ perceived social support from civilian and military sources both correlated negatively with their perceived ostracism (*civilian*: *r* = -.55, *p* < .001; *military*: *r* = -.54, *p* < .001). Thus, our data supported Hypotheses 2a-b.

### Does perceived ostracism explain unique variance in posttraumatic stress?

For Research Question 1a, we conducted a multiple regression analysis. Perceived ostracism explained additional variance in posttraumatic stress symptoms beyond deployment stress and perceived social support (military and civilian): Δ*R*^*2*^ *=* 0.10, *F* (1, 120) = 27.92, *p* < .001.The full results are presented in Table 3. For Research Question 1b, we conducted a relative weights analysis [54] to determine the relative contribution of deployment stress, perceived ostracism, and both types of perceived social support to posttraumatic stress symptoms. Relative weights analysis allows an examination of each predictor’s relative importance considering both the predictor’s direct effect and the predictor’s effect when combined with the other predictors in the regression equation [55]. The analysis was implemented using RWA-Web [56] and the results are presented in Table 4. We calculated rescaled relative weights by dividing each relative weight by the R of the model. The results indicate that perceived ostracism (RRW = 42.41%) explained the most variance in posttraumatic stress symptoms, followed by perceived military support (RRW = 23.47%), perceived civilian support (RRW = 18.48%), and finally reported deployment stress (RRW = 15.64%).

**Table 3:** Incremental variance explained by perceived ostracism over deployment stress, perceived military support and civilian support.

Step	Predictor variable	Β	Δ*R*^2^
1	Deployment Stress Military Support Civilian Support	.30*** -.30** -.29**	.45***
2	Deployment Stress Military Support Civilian Support	.25*** -.18* -.14†	
	Perceived Ostracism	.41***	.10***

†*p* = .078.

*p = .028.

**p = .001.

***p < .001.

**Table 4:** Relative weight analysis.

	Predictor			
	Perceived Ostracism	Deployment Stress	Military Support	Civilian Support
			
RRW	.23	.08	.12	.10
Rescaled RW	42.41%	15.64%	23.47%	18.48%

Notes. R^2^ for the model = .53. RRW = raw relative weights. Rescaled RW = computed by dividing RRW by R^2^ in order to find the percentage of criterion variance attributable to each predictor.

## Conclusions

We provide preliminary empirical evidence supporting the argument that perceived ostracism may be linked to deployment stress and veterans’ mental health concerns [22]. Not only does veterans’ perceived ostracism correlate with frequently occurring psychological problems resulting from deployment (i.e., posttraumatic stress, anxiety and psychological distress), but perceived ostracism explained additional variance in posttraumatic stress and emerged as the most important predictor in the relative weights analysis–almost double the amount predicted by the next highest predictor (i.e., perceived military support). Thus, our data provide an important first step in understanding how perceived ostracism may be related to veterans’ psychological distress post-deployment.

### Limitations and future directions

We have several limitations to consider in contextualizing our results and future directions for research on how perceived ostracism affects veterans post-deployment. First, we used a correlational approach and sampled veterans at only one time point; thus we are limited in any causal interpretations of our data. Future research should employ multi-wave longitudinal survey designs to examine the temporal structure of deployment stress, posttraumatic stress, perceived ostracism, and other types of psychological distress. Presumably veterans develop posttraumatic stress from deployment *before* they perceive ostracism after returning to civilian life, although this is ultimately an empirical question that we cannot answer with our current data. Posttraumatic stress may also create perceptions of ostracism. Additionally, perceived ostracism and posttraumatic stress may create a reciprocal negative feedback loop, exacerbating each other and other types of downstream psychological distress [6; 57].

Second, there are other limitations in our method that need to be considered when conducting future research on this topic. There is the possibility that other non-deployment trauma incidents occurred to participants that may contribute to their scores on the PCL-5 measure, which only asks participants to indicate how much they experienced each symptom within the past month, whereas the deployment stress measure explicitly referenced their pervious deployment. The correlation between the two measures was statistically significant though modest, which may be due to other potentially traumatic events that we could not assess in our survey. However, we did have one item that asked participants how long ago (in months) was their most recent deployment. We re-ran our analyses with this variable included and the data patterns did not change appreciably. The only variable it correlated with significantly (*r* = -.21, *p* = .020) was perceived civilian social support.

We also had a small sample size which limits our ability to investigate complex statistical models. We also only specified in our data collection that participants indicate whether they had been deployed or not; we did not ask a follow-up questions concerning whether they had been in direct combat. Many of the items in our deployment stress measure are specific to combat concerns, but some may be relevant to other deployment contexts. Thus it is possible that the type of deployment context may moderate relations between stress, perceived ostracism, and mental health outcomes. Given the previous studies directly connecting combat-related stressors to mental health outcomes, it is likely that our patterns would be stronger if we restricted our sample to only combat veterans. This is ultimately an empirical question.

Third, we did not assess participants’ beliefs about perceived stigma or self-stigma; each of these components can affect help-seeking [20] and may influence how perceived ostracism relates to psychological health outcomes. Ostracism and other types of interpersonal rejection can cause individuals to experience self-blaming or shame [58–59], and this may be more pronounced among members of stigmatized groups [33, 60–61]. If veterans who are experiencing posttraumatic stress symptoms also self-stigmatize, they may have stronger negative responses to perceived ostracism and intensify the effects of posttraumatic stress on other types of psychological distress [6].

Fourth, our sample has limited generalizability; our participants were previously deployed Illinois National Guard members. Exposure to combat and trauma during deployment can differ by branch of service [5]; perhaps perceptions of social support (civilian or military) and ostracism may also differ. For example, a National Guard member may be the only member of that branch in his or her immediate community, which may reduce the amount of military- and civilian-based social support available, compared with regular Army soldiers who either live on or near a base populated with other soldiers and their families who have similar experiences. Thus, future research should investigate if military branch moderates the relation between these variables on mental health outcomes. Additionally, our sample was mostly male. McGraw [22] discussed how gender might moderate the effects of ostracism on veterans’ mental health, and other research supports this possibility [62].

Finally, there are other measures of psychological distress that can be assessed. We used a general psychological distress index that combined items assessing anxiety (1 item), depression (2 items), and overall positive affect (2 items). We used this index because it was brief and had been used previously to measure psychological distress in a military sample. Future research could use the full measure [48] and examine potential differences between how perceived ostracism relates to anxiety and depression among combat veterans. Additionally, veterans’ exposure to deployment stressors predicts other mental health problems that we did not measure, such as substance abuse and suicide [5, 7, 11–12]. Previous research finds that perceived ostracism and thwarted belonging needs also predict suicidal ideation and related psychological outcomes (e.g., helplessness, meaninglessness; [42, 59, 63]). Indeed, thwarted belonging predicts suicidal ideation above and beyond other risk factors like depression [64]. Perceived burdensomeness also predicts suicidal ideation above and beyond depressive symptoms and interacts with thwarted belonging to increase individuals’ suicidal ideation [64]. If veterans feel burdensome to others in addition to feeling ostracized post-deployment, these feelings may intensify the connection between posttraumatic stress and suicidal behavior.

Our research also has implications for designing and implementing treatment programs for veterans experiencing mental health problems post-deployment. For example, perceived social support from family and friends is inversely correlated with negative posttraumatic cognitions, such as self-blame and expecting future adverse events [65]. These types of cognitive distortions can prolong posttraumatic stress symptoms [66]. Thus, therapists may consider assessing the degree of perceived ostracism involved in these cognitive distortions in individual therapy, and perhaps in group therapy sessions as well to encourage both feelings of inclusion within the group and validation of group members’ perceptions of ostracism from the larger community. Group therapy-based peer support has been found to help veterans cope with trauma by normalizing their experiences [67]; it is likely that normalizing their feelings of ostracism may help reduce the ambiguity that can make ostracism difficult to recover from in other contexts [23], [59]. Overall, our current data suggest that perceived ostracism and related outcomes may be important factors in understanding how to improve deployed veterans’ mental health outcomes upon their return to civilian life and should be considered in future research on treatment and reintegration programs.

## Supporting information

S1 File This is the de-identified, archived data in an SPSS data file. (SAV)
